# Comparison between transcriptomic responses to short-term stress exposures of a common Holarctic and endemic Lake Baikal amphipods

**DOI:** 10.1186/s12864-019-6024-3

**Published:** 2019-09-13

**Authors:** Polina Drozdova, Lorena Rivarola-Duarte, Daria Bedulina, Denis Axenov-Gribanov, Stephan Schreiber, Anton Gurkov, Zhanna Shatilina, Kseniya Vereshchagina, Yulia Lubyaga, Ekaterina Madyarova, Christian Otto, Frank Jühling, Wibke Busch, Lena Jakob, Magnus Lucassen, Franz Josef Sartoris, Jörg Hackermüller, Steve Hoffmann, Hans-Otto Pörtner, Till Luckenbach, Maxim Timofeyev, Peter F. Stadler

**Affiliations:** 1 Institute of Biology, Irkutsk State University, Lenin str. 3, Irkutsk, RUS-664025 Russia; 2 Bioinformatics Group, Department of Computer Science, and Interdisciplinary Center for Bioinformatics, Universität Leipzig, Härtelstraße 16–18, Leipzig, D-04107 Germany; 3 Bioinformatics and Information Technology, Leibniz Institute of Plant Genetics and Crop Plant Research (IPK), Corrensstraße 3, Seeland OT Gatersleben, D-06466 Germany; 4 Plant Genome and Systems Biology, Helmholtz Zentrum München, Ingolstädter Landstraße 1, Neuherberg, D-85764 Germany; 5 Baikal Research Centre, Lenin str. 21, Irkutsk, RUS-664025 Russia; 6 Young Investigator Group Bioinformatics & Transcriptomics, UFZ — Helmholtz Centre for Environmental Research, Permoserstraße 15, Leipzig, D-04318 Germany; 7 ecSeq Bioinformatics GmbH, Sternwartenstraße 29, Leipzig, D-04103 Germany; 8 Inserm U1110, Institut de Recherche sur les Maladies Virales et Hépatiques, 3 Rue Koeberlé, Strasbourg, F-67000 France; 9 Université de Strasbourg, 4 Rue Blaise Pascal, Strasbourg, F-67000 France; 10 Department of Bioanalytical Ecotoxicology, UFZ – Helmholtz Centre for Environmental Research, Permoserstraße 15, Leipzig, D-04318 Germany; 11 Alfred Wegener Institute Helmholtz Centre for Polar and Marine Research, Am Handelshafen 12, Bremerhaven, D-27570 Germany; 12 Max Planck Institute for Mathematics in the Sciences, Inselstraße 22, Leipzig, D-04103 Germany; 13 Department of Theoretical Chemistry, University of Vienna, Währinger Straße 17, Vienna, A-1090 Austria; 14 Facultad de Ciencias, Universidad National de Colombia, Sede Bogotá, Ciudad Universitaria, Bogotá, D.C., COL-111321 Colombia; 15 Santa Fe Institute, 1399 Hyde Park Rd., Santa Fe, NM87501 USA

**Keywords:** Stress response, Baikal, Amhipoda, Heat shock, Heavy metals, Cadmium

## Abstract

**Background:**

Lake Baikal is one of the oldest freshwater lakes and has constituted a stable environment for millions of years, in stark contrast to small, transient bodies of water in its immediate vicinity. A highly diverse endemic endemic amphipod fauna is found in one, but not the other habitat. We ask here whether differences in stress response can explain the immiscibility barrier between Lake Baikal and non-Baikal faunas. To this end, we conducted exposure experiments to increased temperature and the toxic heavy metal cadmium as stressors.

**Results:**

Here we obtained high-quality de novo transcriptome assemblies, covering mutiple conditions, of three amphipod species, and compared their transcriptomic stress responses. Two of these species, *Eulimnogammarus verrucosus* and *E. cyaneus*, are endemic to Lake Baikal, while the Holarctic *Gammarus lacustris* is a potential invader.

**Conclusions:**

Both Baikal species possess intact stress response systems and respond to elevated temperature with relatively similar changes in their expression profiles. *G. lacustris* reacts less strongly to the same stressors, possibly because its transcriptome is already perturbed by acclimation conditions.

**Electronic supplementary material:**

The online version of this article (10.1186/s12864-019-6024-3) contains supplementary material, which is available to authorized users.

## Background

Lake Baikal, located in Eastern Siberia, is the oldest (25-30 million years), by volume largest (23,000 km^3^) and deepest (1643 m) lake on Earth [1]. The ecosystem of Lake Baikal is characterized by vast biodiversity with a high degree of endemism; 80% of the more than 2500 so far described animal species are endemics [2]. This high degree of endemism and the absence of otherwise ubiquitous Palearctic species is probably due to the long, independent evolution of the Baikal endemics [3, 4] that may have highly specialized adaptations to the specific environmental conditions of Lake Baikal, as it is ultra-oligotrophic with low levels of ions and dissolved organic carbon, has high oxygen levels and the low average temperature of 6 ^∘^ C in the littoral zone [5–7]. Ponds in the direct vicinity of Baikal and isolated waters at the shores of the lake are inhabited by species of the Euro-Siberian (Palearctic) fauna, which is completely distinct from the fauna of the open Lake Baikal [1].

The amphipod fauna (Amphipoda, Crustacea) of Lake Baikal is exceptionally species-rich comprising so far described 354 species and subspecies [8]. Lake Baikal amphipods are morphologically and ecologically highly diverse; they inhabit the benthic substrates in all water depths of Baikal, as well as its only outflow, the Angara River. *Gammarus lacustris* Sars, 1863 is common throughout the Holarctic and inhabits very diverse environments, including water bodies in the direct vicinity of Baikal, but the species in general does not intermix with the Baikal endemic gammarid fauna. Endemic littoral Baikal gammarid species, on the other hand, typically do not occur in waters inhabited by *G. lacustris*.

The environmental conditions in the ponds in the vicinity of Baikal and the isolated bays are in many regards highly different to those of the open littoral zone of Lake Baikal, including higher seasonal and daily fluctuations of the water temperature, higher levels of organic substances in those shallow waters, as well as different hydrodynamic regimes [1, 9, 10]. These factors can be an explanation why endemic Baikal gammarids that are highly adapted to a narrow window of environmental conditions are unable to survive in those waters for longer periods. On the other hand, it is a question why species that are ubiquitous in the Palearctic and have adapted to survive in a wide range of environmental conditions are unable to establish stable populations in Lake Baikal. Individuals of *G. lacustris*, for instance, common in water bodies in the direct vicinity of Lake Baikal, may be frequently introduced into the lake, either from the streams entering Lake Baikal, by birds or by humans when used as fish bait.

Our aim with this study was to elucidate the differences between the Palearctic species *G. lacustris* and the most common in the upper littoral of Baikal endemic gammarids *Eulimnogammarus verrucosus* (Gerstfeldt, 1858) and *E. cyaneus* (Dybowsky, 1873) to obtain insights why the Lake Baikal endemic species outcompete the non-Baikal species at Lake Baikal conditions. The peculiarities of Lake Baikal water (see above) may require particular adaptations of aquatic species. Currently, Baikal water is generally highly pristine, and the Lake Baikal endemics may, therefore, be particularly sensitive to chemical water contamination. However, global warming and anthropogenic pollution, e.g. with heavy metals, can change the environmental condition of the Baikal littoral zone. This is why we explored how the gammarids respond to chemical or temperature stress at Lake Baikal water conditions and if there are specific molecular adaptations that provide an advantage to *G. lacustris* over the *Eulimnogammarus* species in the changing environment. We explored the transcriptomes of individuals of the three species upon acclimation to pristine Lake Baikal conditions, i.e., Baikal water at 6 ^∘^ C and from short term (3–24 h) species-specific treatments with cadmium and elevated temperature.

## Results

### Transcriptome assemblies

In order to obtain references for the downstream differential expression analyses, we performed de novo transcriptome assembly for each of the species. The libraries obtained from each type of conditions were included to account for the condition-specific transcripts. Technical characteristics of the assemblies are shown in Additional file 1: Table S1.

**Fig. 1:**
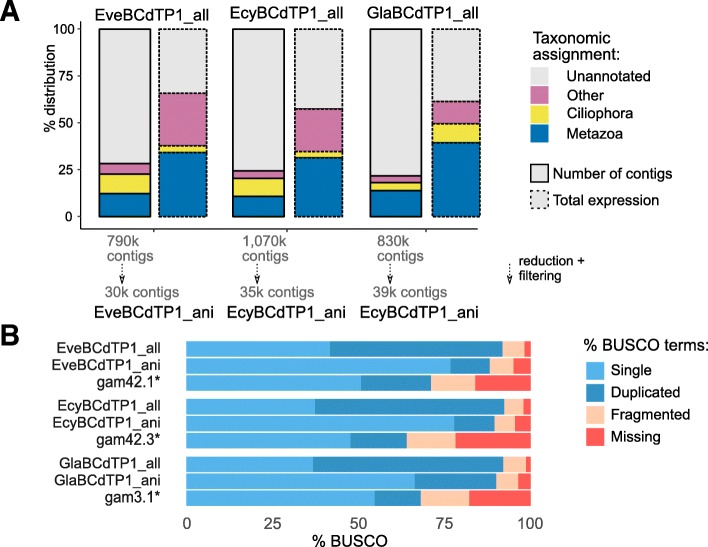
Overview of the assembly composition and completeness. **a**, taxonomic assignment of transcripts. **b**, completeness assession with BUSCO. The assemblies from [12] are shown for comparison (marked with asterisks)

Each transcript was annotated, and its expression was quantified (Fig. 1a). This method reliably identified and taxonomically assigned 25% of the transcripts. For a gross proportion of these transcripts, amounting to about 10% of the different transcript sequences and about 25% of the overall expression, the closest matches were metazoan proteins. Thus, these transcripts can be considered to be originating from gammarid genomes. A considerable number of annotated transcripts amounting to about 5-10% of all transcripts and 5-10% of total expression could clearly be assigned to Ciliophora, which are known to be associated with the gammarids as commensals or symbionts [11]. The remaining transcripts either aligned to proteins from other groups of organisms (presumably symbionts or commensals) or could not be assigned and remained unannotated. Finally, we created a reduced data set with non-redundant sequences most probably originating from the target organism (Fig. 1a; labeled with ani), which would serve as references for mapping reads in the differential expression analysis. As we could not interpret the unannotated transcripts, we did not include them into this reduced set, even though they may represent sequences highly specific for this group of the organisms.

Moreover, we estimated the completeness of our assemblies with BUSCO [13] and compared these values with the published assemblies of the transcriptomes of these species [12]. The assemblies obtained in this work are more complete than the published ones (Fig. 1b).

### Intra- and inter-species variability of transcript abundance

For an estimate to which degree transcript levels differ in all three species and to control for samples that might have been mislabeled (Additional file 1), the raw transcriptome reads obtained from each species were mapped to each transcriptome assembly generated for each of the three species. As expected, in each case the largest number of reads was mapped if the reads and the assemblies were from the same species. The differences in mapping rates of reads to assemblies were quite pronounced (Additional file 1: Figure S1), and thus differential gene expression was determined by mapping the reads to the assemblies of the respective species, except for the two cases (see below).

**Fig. 2:**
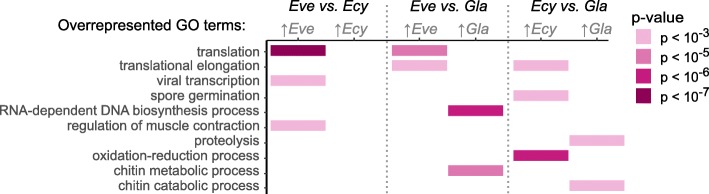
Gene ontology terms that in pair-wise comparisons were enriched in transcripts with higher expression in one species *vs.* the other species

We then compared the control samples (pooled parallel controls of 3- or 24-h treatments) in each pair of species. To account for the difference in mapping rates and reduce the level of noise, we performed each of the three possible interspecies comparisons twice using each of the two assemblies as references. Then we looked for gene ontology (GO) biological process (BP) terms overrepresented in differentially expressed (DE) genes (absolute log_2_ fold change > 3 and adjusted *p*-value < 0.001; unless stated otherwise, the same criteria for differential expression are applied throughout the paper) and selected only those terms that were overrepresented in each of the three species-pairs comparisons regardless of the assembly that was used as a reference and with a significance level with a *p*-value < 0.001 (Fisher’s exact test; Fig. 2).

These comparisons (Fig. 2) show that *E. verrucosus* expressed the transcripts associated with *translation* (GO:0006412) more highly than the other examined species. Furthermore, genes more highly expressed in either *Eulimnogammarus* species relative to *G. lacustris* were enriched in *translational elongation* (GO:0006414)-related transcripts encoding ribosomal proteins and the translation elongation factor 1 (Additional file 2). The differential expression patterns of the transcripts associated with the GO terms *translation* and *translational elongation* in the three species indicate a species-specific protein synthesis activity in the order *E. verrucosus* > *E. cyaneus* > *G. lacustris*.

Other GO terms that were overrepresented in genes over-expressed in *E. verrucosus* in comparison to *E. cyaneus* were *viral transcription* (GO:0019083) including three ribosomal protein-encoding transcripts and *regulation of muscle contraction* (GO:0006937) including transcripts encoding muscular proteins. However, the annotations of all these transcripts are rather vague as they are based on matches of the sequences with sequences from a phylogenetically rather distant taxon, the leech *Helobdella robusta* Shankland, Bissen & Weisblat, 1992 (Additional file 2). Thus, these different expression patterns do not clearly indicate gene expression differences between the studied amphipod species. Instead, they may indicate differing abundances of parasites in the examined animals; leeches are known parasites of Baikal amphipods [14].

GO terms overrepresented in transcripts with higher expression in *E. cyaneus* in comparison to *G. lacustris* were *spore germination* (GO:0009847) and *oxidation-reduction process* (GO:0055114). Both terms comprised transcripts encoding 14-3-3 proteins; the term *oxidation-reduction process* also contained transcripts encoding hemocyanin subunits, dehydrogenases and oxidases.

The GO term *RNA-dependent DNA biosynthetic process* (GO:0006278) was overrepresented in transcripts with higher expression in *G. lacustris* compared to *E. verrucosus*. The respective transcripts comprised mostly RNA-directed DNA polymerases from different transposable elements.

Other GO terms enriched in genes with lower expression in both *Eulimnogammarus* species compared to *G. lacustris* were *proteolysis* (GO:0006508) with transcripts predominantly encoding proteases and the two similar biological processes *chitin catabolic process* (GO:0006032) and *chitin metabolic process* (GO:0006030) that also included chitin-degrading enzymes.

To estimate the noise level in our data, we performed a comparison of expression profiles in two control groups (3-h against 24-h parallel controls) for each species with same-species mapping. The number of genes differentially expressed at the chosen threshold levels (absolute log_2_ fold change value > 3 and adjusted *p*-value < 0.001) was as low as two genes for *E. verrucosus*, and no genes were differentially expressed in the other two species. The observed agreement in the two control groups ensures that most differences between the experimental and control conditions should be true positives.

### Responses to thermal stress

**Fig. 3:**
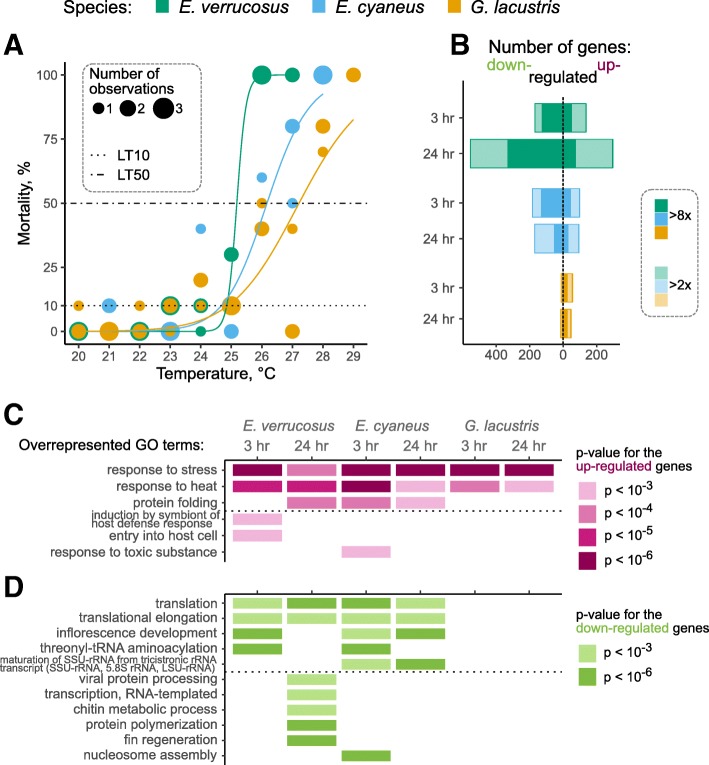
Temperature sensitivities of the different amphipod species and their transcriptional responses to elevated temperature. **a**, % mortalities of the studied species at different temperatures. The point size is proportional to the number of data points for the same species located at the same coordinates; this solution was chosen to avoid overplotting. **b**, numbers of up- and down-regulated genes in each species after 3-h and 24-h exposures to the species-specific LT10. The full list of DE genes and the corresponding fold change and *p*-values are available from Additional file 3. **c**, GO terms enriched in the transcripts up-regulated under acute heat shock. **d**, GO terms enriched for the transcripts down-regulated under these conditions. Only GO terms enriched with a *p*-value of at most 0.001 and including at least two differentially expressed transcripts are shown

*Lethal temperatures:* Temperature sensitivities of the studied species were estimated by determining the species-specific lethal temperatures. Individuals of the three amphipod species were incubated at temperature stress conditions, i.e., at water temperatures between 20 and 29 ^∘^ C for 24 h, and dead individuals were counted. The lethal temperatures for 50% of the individuals (LT50) differed considerably between the species ranging from 25.2 to 27.2 ^∘^ C (Fig. 3a) and mirroring the differences in temperature preferences of the species determined previously [15, 16]; however, the 10% mortality temperature (LT10) turned out to be very similar for all species (24.9, 24.6 and 24.6 ^∘^ C for *E. verrucosus*, *E. cyaneus*, and *G. lacustris*, respectively). For *E. verrucosus* the range of the temperatures causing no and 100% lethality was with 24–26 ^∘^ C comparatively narrow, whereas this range was wider for *E. cyaneus* with 23–28 ^∘^ C and for *G. lacustris* with 23–29 ^∘^ C. Thus, although LTx values of the different species were in the lower temperature range similar, the higher LTx values varied more across the species due to the different slopes of the regression curves (Fig. 3a). The species-specific LT10 values were chosen for the temperature stress treatments in the studies of the transcriptomic responses of the amphipod species to thermal stress.

*Expression responses to temperature stress*: The numbers of differentially expressed transcripts varied across the different amphipod species upon 3- and 24-h exposures to LT10 over one order of magnitude (Fig. 3b; Additional file 3). The more temperature-sensitive species, i.e., the species with lower LT50, showed higher numbers of responsive genes (Fig. 3a, b). To obtain an indication which biological processes were affected by temperature stress across the different species, we identified GO terms overrepresented in up-regulated or down-regulated transcripts (Fig. 3c, d, Additional file 4).

All species showed up-regulation of very similar groups of genes associated with the GO terms *response to stress* (GO:0006950), *response to heat* (GO:0009408) and *protein folding* (GO:0006457). These GO terms comprise transcripts encoded by heat shock protein (*hsp*) genes *hsp60*, *hsp70* and *hsp90*; *dnaJ* (*hsp40*); small heat shock proteins such as *hsp20* (Additional file 4); and one polyubiquitin gene. Raised temperature (i.e., heat shock) is known to induce representatives of all heat shock protein gene families [17].

Accordingly, genes up-regulated in response to elevated temperature were enriched in molecular function (MF) GO terms *ATP binding* (GO:0005524) and *unfolded protein binding* (GO:0051082). Overrepresented cellular component (CC) GO terms varied across the different species; except for the LT10 (24 h) treatment of *G. lacustris*, GO terms related to cell surface were overrepresented in all species in all treatments (Additional file 4). However, as this GO term comprises several heat shock protein transcripts that generally were induced in the LT10 treatments, the CC overrepresentation may be the consequence of diverse functions of heat shock proteins. Taken together, these results show that a variety of heat shock genes induced by elevated temperatures exists in the studied amphipods.

Other overrepresented GO were *induction by symbiont of host defense response* (GO:0044416), *entry into host cell* (GO:0030260) and *response to toxic substance* (GO:0009636). All of them included *hsp70* genes; this result most probably reflects the multifaceted functional possibilities of these proteins. The latter term also contained a glutathione S-transferase (*gst*) transcript. Different transcripts encoding glutathione S-transferases were up-regulated (more than sixty-fold) or down-regulated (approx. twenty-fold) in *E. cyaneus* in the 3-h LT10 treatment, as well as down-regulated (approx. fourteen-fold) in the 24-h LT10 treatment in *E. verrucosus*. Among other antioxidant enzymes, a transcript presumably encoding a copper/zinc superoxide dismutase was down-regulated in 3 h and 24-h LT10 treatments in *E. verrucosus*.

Effects of elevated temperature on the activities of key metabolic enzymes cytochrome C oxidases (COX), citrate synthases, lactate dehydrogenases, pyruvate kinases, 3-hydroxyacyl-CoA dehydrogenases, and glutamate dehydrogenases in amphipods have been studied earlier [18]. Thus, here we attempted to determine expression changes of the respective genes.

Among key metabolic enzymes, we searched for cytochrome C oxidases, citrate synthases, lactate dehydrogenases, pyruvate kinases, 3-hydroxyacyl-CoA dehydrogenases, and glutamate dehydrogenases. The putative *cox3* transcript was induced four-fold in *E. cyaneus* from the 24-h LT10 treatment and eight-fold and six-fold in *E. verrucosus* from the 3- and 24-h LT10 treatments, respectively. In contrast, another transcript annotated as *cox2* more than ten-fold down-regulated in *E. verrucosus* from the 24-h LT10 treatment. No other *cox*-related transcripts was differentially expressed in any of the studied species, even though other *cox* contigs were present in all assemblies.

The common biological processes inhibited by heat shock in both *Eulimnogammarus* species included *translation* (GO:0006412) and *translational elongation* (GO:0006414). Overrepresentation of these terms was due to genes encoding ribosomal proteins of both subunits, eukaryotic elongation factor 1 and a transcript for a potential Cu-Zn superoxide dismutase. The terms *inflorescence development* (GO:0010229) and *maturation of SSU-rRNA from tricistronic rRNA transcript (SSU-rRNA, 5.8S rRNA, LSU-rRNA)* (GO:0000462) were also overrepresented due to ribosomal protein transcripts, particularly those encoding RPL27 in the former and S8 in the latter case. The term *threonyl-tRNA aminoacylation* (GO:0006435), which was overrepresented in *E. verrucosus* and *E. cyaneus*, results from two transcripts in each case, both of which have an unannotated protein XP_001618065 from *Nematostella vectensis* Stephenson, 1935. However, more in-depth blast search revealed that only the best blast hit belong to the aforementioned coral species, while the other results are elongation factors from algae. As these transcripts might have been contaminants, we did not analyze them further.

Some of the groups were characteristic only for one combination of species and exposure time. The term *nucleosome assembly* (GO:0006334), which was characteristic only for *E. cyaneus* after 3-h exposure, was represented by several histone-encoding transcripts. The terms *protein polymerization* (GO:0051258) and *fin regeneration* (GO:0031101) were only characteristic for *E. verrucosus* after 24-h exposure to elevated temperature. They included tubulin-encoding and cathepsin-encoding transcripts, respectively. Finally, the term *chitin metabolism* (GO:0006030) indicated a lower expression of chitinases in heat-exposed *E. verrucosus*.

The top MF and CC GO terms enriched in genes down-regulated in the *Eulimnogammarus* species in response to heat shock were also connected to ribosomes and translation, such as *structural constituent of ribosome* (GO:0003735), *rRNA binding* (GO:0019843), *translation elongation factor activity* (GO:0003746), *GTPase activity* (GO:0003924), *ribosome* (GO:0005840) and *cytosolic large ribosomal subunit* (GO:0022625).

In summary, all responses common to at least two combinations of species and conditions are translation-related. Interestingly, these responses were only recorded in the Baikal *Eulimnogammarus* species, but not in the Holarctic *G. lacustris*. However, the absence of response in *G. lacustris* could be explained simply by the small number of DE transcripts (Fig. 3b). To account for this possibility, we relaxed the stringency of our filtering (at least two-fold decrease in expression with adjusted *p*-value < 0.05) and looked for transcripts annotated as potential ribosomal proteins in *G. lacustris*. Indeed, we found two transcripts annotated as L37 and L23 proteins down-regulated in *G. lacustris* after 3 h of heat shock; no difference in the amount of ribosomal protein-encoding transcripts could be detected after 24 h of heat shock in this species. Thus, the translation-related response is also possible in *G. lacustris* in these conditions but is certainly much weaker.

### Genes regulated in response to cadmium

The toxic sensitivities of the examined amphipod species to cadmium were determined earlier. For examining the transcriptomic responses to CdCl_2_ exposure, individuals of each studied species were exposed to the species-specific LC10 (24 h) (2.9, 3.7 or 3.2 *μ*M for *E. verrucosus*, *E. cyaneus* and *G. lacustris*, respectively [19]).

**Fig. 4:**
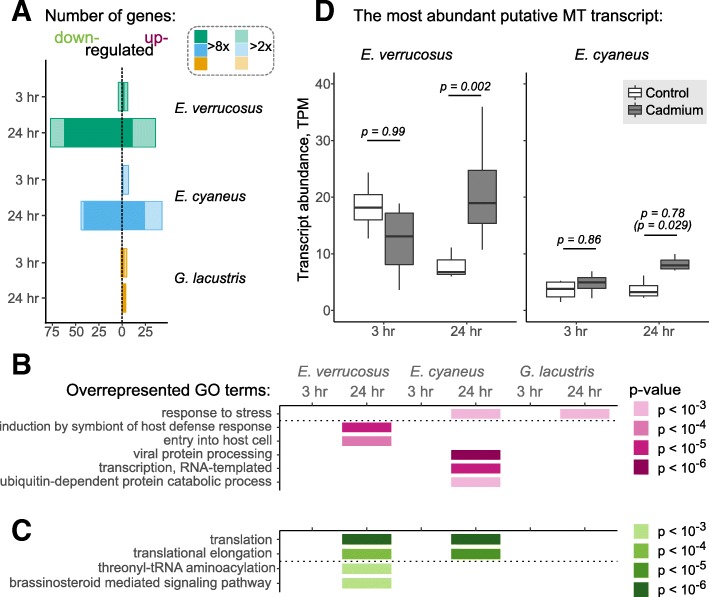
Transcriptional responses to cadmium treatment. **a**, numbers of up- and down-regulated genes in each species after 3-h and 24-h exposures to the species-specific LC10. **b**, GO terms enriched in the transcripts up-regulated under cadmium treatment. The full list of DE genes and the corresponding fold change and *p*-values are available from Additional file 3. **c**, GO terms enriched in the transcripts down-regulated under these conditions. Only GO terms enriched with a *p*-value of at most 0.001 and including at least two differentially expressed transcripts are shown. Only GO terms enriched with a *p*-value <0.001 and including at least two differentially expressed transcripts are shown. **d**, summary of expression for the two most abundant MT-like transcripts. The *p*-values shown on top of the boxplots come from the DESeq2 analysis, except for the value in parentheses calculated with the Mann-Whitney test

By the overall number of genes all samples can be divided into two groups. While *E. verrucosus* and *E. cyaneus* show a significant response after 24-h exposure (> 60 DE genes), in all the other cases the response is very subtle (< 5 DE genes; Fig. 4a; Additional file 3).

The diversity of overrepresented GO terms under cadmium treatment is greater and less uniform than that observed under heat shock (Fig. 4b, c, compare with Fig. 3c, d). The only common GO term enriched in the genes up-regulated under cadmium treatment is the *response to stress* (GO:0006950), which includes mostly the transcripts annotated as putative 70 kDa heat shock protein family members (Additional file 4). In addition, in the case of *E. cyaneus* this group also includes a polyubiquitin-C. The remaining terms unique for *E. cyaneus* were also based on ubiquitin-related degradation and included various transcripts potentially encoding polyubiquitin-C, as well as those encoding ubiquitin ligase-like proteins. The terms unique for *E. verrucosus*, namely *induction by symbiont of host defense response* (GO:0044416) and *entry into host cell* (GO:0030260), united the same *hsp70* transcripts as in response to high temperature. The only transcript encoding an antioxidant enzyme and up-regulated under this treatment was a glutathione S-transferase transcript in *E. cyaneus* after 3-h treatment.

Heavy metals are bound by specific proteins, metallothioneins [20]. Metallothionein (MT)-encoding sequences have been reported from the genome and transcriptome of *Hyalella azteca* Saussure, 1858 [21] an the transcriptome of another amphipod species, *Grandidierella japonica* Stephensen, 1938 [22]; the abundance of these sequences was approximately two-fold higher in response to zinc exposure ([21, 22]) and even 16-fold higher in response to cadmium exposure ([21]). Therefore, we looked for transcripts that potentially encoded MT in our full datasets and found from 3 (in *G. lacustris*) to 13 (in *E. cyaneus*) such transcripts in our assemblies (Additional file 6). However, none of the transcripts was differentially expressed according to our standards, even though the majority of transcripts in Baikal species had higher median abundance values under cadmium treatment (Additional file 1; Fig. 4d). Moreover, most of the transcripts had low expression: only six transcripts in *E. cyaneus*, one transcript in *E. verrucosus* and no transcripts in *G. lacustris* had abundance higher than one transcript per million (Additional file 1: Figure S3). Expression levels of the two most abundant transcripts are shown in Fig. 4d. The predicted protein sequences encoded by these transcripts are identical, and the most similar sequence in the NCBI nr database turned out to be the MT of a deep-sea vent brachyuran crab *Allograea tomentosa* Guinot, Hurtado & Vrijenhoek, 2002. The sequence of *A. tomentosa* was only 53% identical to those of *Eulimnogammarus* sp., but all the Cys residues were conserved within the three proteins (Additional file 1: Figure S3), suggesting that these transcripts indeed encode metallothionein-like proteins. These transcripts were not differentially expressed under cadmium treatment according to our criteria. The fold change was not consistent between the two time points, and while the expression change observed in *E. verrucosus* upon 24-h treatment had an associated *p*-value of 0.002, in *E. cyaneus* it was 0.78, even though independent testing of these values without correction for multiple testing would return a *p*-value of 0.029. Thus, the questions of specificity of the putative products of these transcripts and regulation of their expression in response to heavy metals remain open.

The GO terms overrepresented among down-regulated genes were mostly the same as featured in the response to elevated temperature (Fig. 3d), even though the diversity was lower (Fig. 4c), and included the same genes (Additional file 4). The term *brassinosteroid mediated signaling pathway* (GO:0009742) included two 14-3-3 protein transcripts.

### Comparison between transcripts responding to temperature and cadmium

**Fig. 5:**
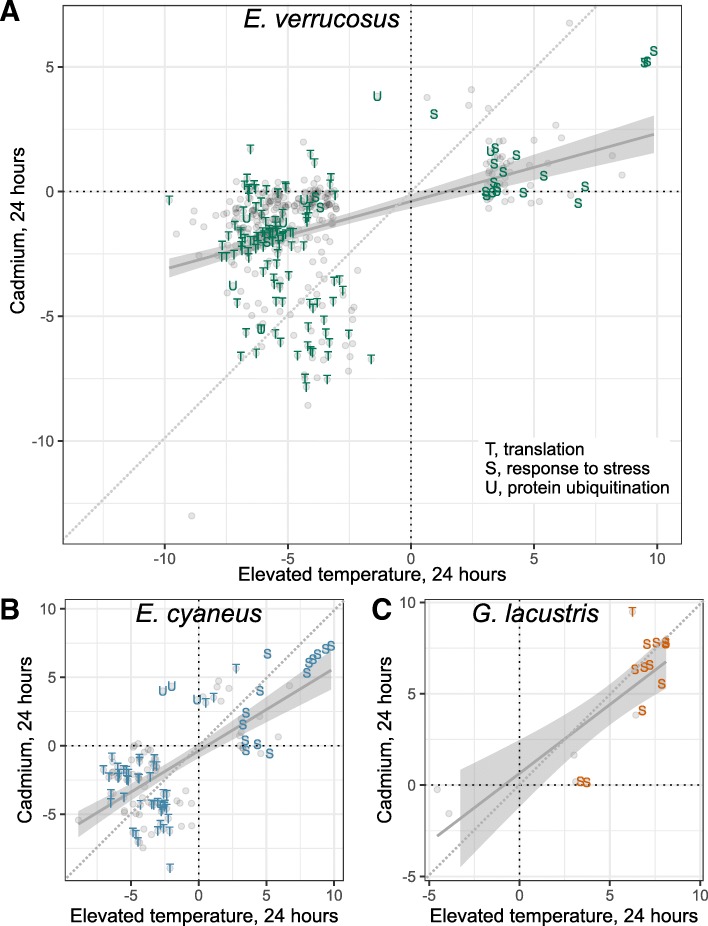
Comparison of differentially expressed genes between heat shock and heavy metal stress. Each point corresponds to a gene which was differentially expressed in at least one of the conditions indicated along the axes. Along each axis are log_2_ fold changes relative to the control group. T, translation-related; S, response to stress or response to heat; U, ubiquitin-proteasome-related system; P, proteolysis. Shown are automatically fitted linear regression models

We subjected the animals to two stressors and used GO terms to compare responses between different species. However, tracing reaction of individual transcripts rather than GO terms comparison between their responses would tell us if the same cellular stress response systems control them—or there were condition-specific DE transcripts associated with the same GO term. To test these hypotheses, we built scatterplots for all genes that were differentially expressed in response to at least one of the treatments after 24-h exposure (Fig. 5) and also fitted linear regression models to describe common tendencies. The regression lines are closer to the horizontal axis, thus showing that the response to elevated temperature is generally more pronounced than the response to cadmium treatment (Fig. 5), especially in the case of *E. verrucosus*. This result was corroborated by comparison of the numbers of DE genes (see above).

Moreover, there were temperature-specific stress response transcripts in each species (those labeled with *S* and being close to zero on the vertical axis but positive on the horizontal axis). Furthermore, while some translation-related transcripts in *E. verrucosus* reacted equally well to temperature and cadmium, some others only reacted to temperature. Interestingly, in the two *Eulimnogammarus* species there were cadmium-specific ubiquitin-proteasome system components (Fig. 5a, b; Additional file 5).

## Discussion

In this work, we compared the transcriptomic stress responses in three amphipod species; two, *E. verrucosus* and *E. cyaneus*, are endemic to Lake Baikal, while *G. lacustris* occurs in the Holarctic and can be regarded a potential invader species to Baikal. Animals were either kept in the conditions simulating those of the Baikal littoral (control), subjected to the temperature stress (LT10) or chemical stress (LC10 CdCl_2_). This experimental setup allowed us to compare different species, as well as responses to different stressors.

### Transcriptional profiles clearly distinguish species

To compare transcriptional profiles between species, we analyzed interspecies differences in the control samples. As the species have quite different transcript sequences, the results of this comparison were noisy. Nevertheless, some results were robust (Fig. 2). We were able to conclude that *E. verrucosus* has the highest expression activity of translation-related genes. This may reflect intrinsic characteristics of protein synthesis in each species but may also be related to the exposure conditions, as the temperature was comparatively close to the optimal for *E. verrucosus* and most distant from the optimal or preferred for *G. lacustris* [15, 16].

The non-Baikal species *G. lacustris* also had other hallmarks in its transcription profile. It had higher expression of transcripts encoding RNA-directed DNA polymerases from various mobile elements, including Ty3-like and jockey-like (Additional file 2) compared to *E. verrucosus*. As we did not have control samples of *G. lacustris* from its native habitat, we cannot tell if the increased transposon activity is characteristic for the species or is triggered by the acclimation conditions and can only speak about differences between *G. lacustris* and *E. verrucosus* acclimated to the same conditions. We are not aware of studies exploring the differential expression of transposable elements in amphipods, but such analysis has been performed for an insect, *Locusta migratoria* ssp., and revealed changes between life stages [23], but we only studied the adult form. Thus, the interspecies difference in transposon activity can hardly be interpreted with the available data and at the current level of knowledge, but it can provide hypotheses to test in further research.

Another characteristic feature of *G. lacustris* was over-expression of protease- and chitin catabolism-related transcripts (Additional file 2). The expression of chitinase genes in crustaceans generally peaks at pre-molt stages [24]. This result might indicate induction of molting in *G. lacustris*, which may have been triggered by acclimation conditions.

At the same time, transcripts related to oxidation-reduction processes were characterized by higher expression in *E. cyaneus* than in *G. lacustris*, while expression of this group of transcripts in *E. verrucosus* is supposedly intermediate, as it was not significantly different from either species. According to routine oxygen consumption measurements [25], *E. verrucosus* had the lowest metabolic rate, while these values for the other species were comparable. A possible explanation for this discrepancy between the physiological measurements and transcriptional profiles could be that *G. lacustris* uses additional energy for pre-molting processes, as signified by higher expression of protease-related and chitin metabolism-related genes.

### Transcriptional stress response has both species- and stressor-specific features

All species showed clear stress response, and this response (assessed as the number of DE genes) was least pronounced in *G. lacustris*, intermediate in *E. cyaneus* and most prominent in *E. verrucosus*. Moreover, the response to cadmium treatment was generally weaker than the response to the elevated temperature (compare Fig. 3b with Fig. 4a). We did not find specific molecular adaptations to these stressors that would differ between Baikal endemics and the generalist species *G. lacustris*, even though we cannot exclude the possibility that some specific transcripts not annotated so far could create such a difference.

Exposure of the species to two different stressors clearly reveals similar response in the two Baikal species, which comprised increased expression of known stress response transcripts, mostly those encoding molecular chaperones, and decreased expression of translation-related transcripts, mostly those encoding ribosomal proteins (Figs. 3 and 4). The heat shock factor (Hsf) is traditionally viewed as the master transcriptional regulator of heat shock response [26], even though newer data suggest not only that Hsf1 may regulate other genes but also that not all heat stress-responsive genes are regulated by Hsf1 [27]. Arthropod species have only one gene encoding this protein, and the activity of Hsf1 is primarily regulated at the post-transcriptional level by oligomerization and phosphorylation [28]. Our data are consistent with this notion: each of the transcriptomes contained transcripts with best hits annotated as Hsf1 in other arthropod species, but none of them were differentially expressed. As we had only mRNA sequences but not the corresponding genomic DNA sequences at our disposal, we could not directly assess the correlation between changes in gene expression and presence of putative Hsf1 binding sites in the promoters. Further analysis based on genomic data might shed more light onto the mechanism behind stress-induced changes in gene expression.

Inhibition of translation is a well-known cellular-level response to multiple stressors. This pathway is mediated by phosphorylation of eukaryotic translation initiation factor 2 *α*, and among the downstream targets of this protein is the transcription factor Atf4 [29], which can in turn activate the synthesis of some inhibitor of ribosomal protein gene expression. The shutdown of ribosomal protein gene transcription under stress in the yeast *Saccharomyces cerevisiae* Meyen ex E.C. Hansen, 1883 is a long-known phenomenon [30] explained by target of rapamycin (TOR) kinase complex-mediated action of multiple regulators of transcription [31]. This pathway has not been studied in detail in invertebrates, but there are examples of decreased transcription of ribosomal genes in response to temperature stress in insects [32], corals [33], or *Daphnia pulex* Leydig, 1860 [34]. This response can also be mediated via the TOR kinase, but its universality and the mechanism need additional research.

In this work, we subjected the animals to two different stressors but found that gene expression changes are quite similar (Fig. 5). This result allows us to conclude that Baikal amphipods have intact stress response systems, which reacted to either stressor. However, changes in the expression of some transcripts were stressor-specific. The existence of heat-shock specific *hsp* transcripts might mean that there is more than one system regulating the transcription of these genes. At the same time, some ubiquitin-proteasome-related transcripts were only induced by cadmium treatment but did not react to temperature, also suggesting the existence of a specific mechanism activated by this heavy metal. In addition, we found some metallothionein-related transcripts but did not find clear induction in response to the treatment. The specificity of induction conditions and diversity of these genes also require additional investigation.

The Holarctic species *G. lacustris* shows a similar *hsp* response as the Baikal species, but the overall amplitude of expression changes is lower, and translation-related response is also much weaker, even though the treatments caused the same organismal-level adverse effect (10% lethality) for all investigated species. However, prior to these experiments the *G. lacustris* individuals were acclimated under the conditions of Lake Baikal, i.e. a temperature of 6 ^∘^ C and in Baikal water. It is possible that the animals already experienced inhibition of ribosomal protein gene expression, and we did not see any response because further down-regulation was not possible. Alternatively, it is possible that the observed characteristics of the transcription profile are inherent for these species. As we did not have a pre-acclimation control or control acclimated in the water from *G. lacustris* habitat, we could not directly test these hypotheses. Taken together with the results of the interspecies comparison, these results nevertheless allow us to suggest that inhibition of ribosomal protein gene expression is associated with stress response and that gene expression in *G. lacustris* is already perturbed by acclimation to the conditions of Lake Baikal littoral. Thus, it is possible that the adaptation potential of the former species is not much wider than for Baikal endemics: even though *G. lacustris* inhabits water bodies with cold water, it is possible that under Baikal conditions it survives for some time but is not fully adapted to them, and thus cannot invade the Lake Baikal littoral in its current state.

## Conclusions

Here we studied the transcriptomic responses of two endemic Baikal amphipod species and a Holarctic species to two proteotoxic stressors, elevated temperature and heavy metal (cadmium) exposure, at species-specific 10% mortality levels, in comparison to the control conditions matching the conditions of Lake Baikal littoral. The results indicate that both Baikal species possess intact stress response systems and respond to the stressors with similar changes in their transcription profiles, while the Holarctic species *G. lacustris* has a less prominent response to both stressors. This result might be explained if Baikal species have specific metabolic adaptations to low temperatures and water composition, while the Holarctic species is not fully adapted to the current conditions of Lake Baikal littoral, and its transcriptome is already perturbed by acclimation.

## Materials and methods

### Animals and experiments

*Eulimnogammarus verrucosus* (Gerstfeldt, 1858) and *Eulimnogammarus cyaneus* (Dybowsky, 1874) are amphipod species endemic to Lake Baikal. *Gammarus lacustris* Sars, 1863 inhabits various water bodies throughout the Northern hemisphere [8]. Amphipods were collected in August 2013 with a hand net at depths of 0.5–1.5 m. *Eulimnogammarus* sp. individuals were collected in the Baikal littoral near the Bolshie Koty village (south-west coast of Baikal, 51 ^∘^ 54’11.67”N 105 ^∘^ 4’7.61”E); water temperature at sampling varied within 12–16 ^∘^ C. *G. lacustris* individuals were collected in Lake 14 in the vicinity of Bolshie Koty (51 ^∘^ 55’14.39”N, 105 ^∘^ 4’19.48” E); the temperature at sampling varied within 10–12 ^∘^ C. All animals were pre-acclimated for one week at 6±1 ^∘^ C in well-aerated Baikal water. During acclimation, the amphipods were fed ad libitum with dried and ground invertebrates and algae from the Baikal littoral; water was exchanged every three to four days. No mortality was observed during acclimation.

The lethal temperatures were determined by incubating ten individuals of each species, sampled at the same time, in thermally insulated aquaria with well aerated Baikal water at a temperature range from 20 to 29 ^∘^ C with one-degree increment. Three independent replicates, i.e. aquaria containing ten individuals, were used for each species and temperature, except for the two cases of extreme temperatures (*E. verrucosus*) at 27 ^∘^ C and *G. lacustris* at 28 ^∘^ C with two replicates each. The lethality was recorded after 24 h, and the regression analysis was carried out in GraphPad Prism 7.0a with the Weibull model. LT10 values were calculated according to the regression mortality curve for each species.

Parallel controls were kept at 6 ^∘^ C (acclimation temperature) in clean Baikal water. Experimental individuals were exposed at the calculated 24-h LT10 temperatures or in water with either of tested substances. These substances included CdCl_2_ in the final (nominal) concentration of 2.9, 3.7, or 3.2 *μ*M, which corresponded to 24-h LC10 [19], acetone (0.1*%*) or phenanthrene (1 mg/L, dissolved in acetone; final concentration of acetone 0.1%). After 3 or 24 h of exposure, as indicated, the animals were quickly frozen in liquid nitrogen. In addition, groups of animals were kept at constantly rising temperature (0.8 ^∘^ C/day) from 6 to 24.4 ^∘^ C and frozen at 12.4, 18, and 24.4 ^∘^ C with parallel control groups kept at 6 ^∘^ C under the whole time of the experiment. The results of acetone, phenanthrene, and constantly rising temperature exposures were used to obtain de novo transcriptome assemblies, but differential expression analysis was not presented in the current study. Three to four biological replicates (animals from different aquaria) were analyzed for each species and condition combination (control, LT10 or cadmium treatment).

In addition, the water after 24-h Cd treatment was preserved by freezing (−20 ^∘^ C), and the amount of Cd in the water was later measured by inductively coupled plasma mass spectrometry using an Agilent 7700 mass spectrometer (Agilent Technologies) in two replicates for each species. The average concentration of Cd measured at the end of the exposure was never below 75% of the nominal concentration in the beginning of the exposure (2.2 *vs.* 2.9, 3.1 *vs.* 3.7, and 2.5 *vs.* 3.2 *μ*M for the measured and nominal concentrations for *E. verrucosus*, *E. cyaneus* and *G. lacustris*, respectively).

### RNA extraction, library preparation, and next-generation sequencing

Total RNA for sequencing was extracted from single individuals of *E. verrucosus*, a pool of four to five individuals in the case of *E. cyaneus* (up to 700 mg) or pools of three individuals of *G. lacustris*. Frozen amphipods were homogenized in 1 ml of Qiazol Reagent (Qiagen) using an MM400 homogenizer (Retsch). RNA isolation from the aqueous phases was performed using a Qiacube (Qiagen) with the miRNeasy kit (Qiagen), and mRNA enrichment was performed with the Oligotex mRNA Mini Kit (Qiagen). RNA quality control was performed with the RNA 6000 Nano Kit on an Agilent Bioanalyzer 2100. mRNA libraries were prepared with the ScriptSeq^TM^ v2 RNA-Seq Library Preparation Kit (Epicentre, # SSV21124) and sequenced with Illumina HiSeq2000 (48 samples per flow cell). Raw sequencing data are available from NCBI under the Bioproject accession PRJNA505233.

### Read quality control and de novo transcriptome assembly

Quality control for the reads was performed with fastQC [35] v0.11.5, and results were summarized with MultiQC [36] v1.2. Illumina universal adapter sequence was found at the end of up to 10–20% sequences, probably because the insert was below the expected length. These adapter sequences were trimmed with trim_galore [37] v0.4.1. Moreover, up to approximately 27% of the forward reads and approximately 20% of the reverse reads were assigned to overrepresented sequences. These sequences were analyzed with the NCBI BLAST web interface [38] and assigned to ribosomal RNA sequences. To filter out these sequences, a custom database of rRNA sequences of gammarid amphipods Additional file 7 was created. All reads were aligned to this database with bowtie2 [39] v2.1.0 with the –very-sensitive-local parameter enabled, and the clean reads (e.g., those not aligned to any ribosomal RNA sequence) were dumped into new files with the –un-conc-gz option. At the next step, the clean reads were subjected to read error correction with RCorrector [40] (cloned from Github on Dec 18, 2017). Finally, de novo transcriptome assembly was performed with Trinity [41] v2.4.0 with the –SS_lib_type FR parameter enabled to utilize the strand specificity of the libraries.

Technical characteristics of the assemblies are shown in Additional file 1: Table S1. These assemblies will be referred to with the first letter of the genus, two letters of the species name, conditions used and all (e.g., EveBCdTP1_all).

### Assembly quality control, annotation, and filtering

Quality control of the assemblies was performed with TransRate [42] v1.0.3. Assembly completeness was estimated with BUSCO [13] v3.0.2.

Each transcript was annotated with the blastx analog of the accelerated blastp-like program diamond [43] v0.9.10.111 against the NCBI non-redundant protein sequence database of Oct 10 2017. It allowed us to assign the best protein hit for transcripts (contigs) and also provide taxonomic assignations based on this best hit. Then, redundant sequences were removed with the Evidentialgene pipeline [44], and only the contigs with the best diamond hit to a sequence belonging to a metazoan species were selected. This reduced data set was labeled with ani (e.g., EveBCdTP1_ani; Fig. 1a) and served as references for mapping reads in the differential expression analysis.

Additional annotation, including GO terms mapping with Blast2GO [45], was performed with the FunctionAnnotator web server [46].

### Differential expression

Transcript quantification was performed with salmon [47] v0.9.1, and subsequent analysis was performed with DESeq2 [48] v1.14.1 and tximport [49] v1.2.0 packages for R [50]. The *p*-values were adjusted according to the Benjamini and Hochberg procedure [51], which is the default option in DESeq2 [48]. Only transcripts with the adjusted *p*-value below 0.001 and the absolute logarithm of fold change above 3 (i.e., 8-fold difference) were considered differentially expressed unless stated otherwise. GO term overrepresentation analysis was implemented with the topGO package [52] v2.34.0 for R. In-house R scripts for data analysis are available at github, see [53].

## Additional files

Additional file 1 Table S1: Characteristics of the assembly.
Text S1: Checks for mislabeled samples.
Figure S2: Transcripts of glutathione S transferase gene family.
Figure S3: Putative metallothionein (MT) transcripts. (PDF 937 kb)

Additional file 2 GO terms overrepresented in transcripts that were differentially expressed between species. Terms that are robust regardless of the assembly used as a reference are shown in bold. Annotated: the number of transcripts annotated with the corresponding GO term. Significant: the observed number of DE transcripts annotated with the corresponding GO term. Expected: the expected number of DE transcripts. (XLSX 176 kb)

Additional file 3 List of transcripts that were differentially expressed in response to elevated temperature or cadmium. (XLSX 236 kb)

Additional file 4 GO terms overrepresented in transcripts that were differentially expressed in response to elevated temperature or cadmium. For explanations of the column names please see the caption for Additional file 2. (XLSX 62 kb)

Additional file 5 Comparison of transcripts differentially expressed in response to temperature and/or cadmium treatments. (XLSX 51 kb)

Additional file 6 Sequences and characteristics of metallothionein-like transcripts. (XLSX 13 kb)

Additional file 7 Ribosomal RNA sequences used to filter reads. (FA 20 kb)

## Data Availability

The datasets generated and/or analyzed during the current study have been submitted to the NCBI database under the BioProject PRJNA505233, including the raw sequencing data in the SRA database, the transcriptome assemblies in the GenBank database under the accession numbers GHHK00000000 (*E. verrucosus*), GHHW00000000 (*E. cyaneus*), and GHHU00000000 (*G. lacustris*), and the differential expression data are available in the GEO repository under the accession number GSE129069.

## Abbreviations

BP: Biological process
CC: Cellular component
DE: Differentially expressed
GO: Gene ontology
Hsf: Heat shock factor
hsp: Heat shock protein
LC*x*: Lethal concentration for *x*% of the population
LT*x*: Lethal temperature for *x*% of the population
MF: Molecular function
MT: Metallothionein
