# Supplementary material for: Comparison between transcriptomic responses to short-term stress exposures of a common Holarctic and endemic Lake Baikal amphipods
Source: BMC Genomics. 2019 Sep 13;20:712. doi: 10.1186/s12864-019-6024-3 (PMC6743106; doi:10.1186/s12864-019-6024-3)
Supplement: Supplementary file 1 — Table S1: Characteristics of the assembly. Text S1: Checks for mislabeled samples. Figure S2: Transcripts of glutathione S transferase gene family. Figure S3: Putative metallothionein (MT) transcripts. (PDF 937 kb) [file 12864_2019_6024_MOESM1_ESM.pdf]

Additional Results

Assembly statistics

Table S1: characteristics of the assembly. Shown are technical characteristics of the complete and reduced transcriptome assemblies according to TransRate [42]. The explanation of the terms can be found at http://hibberdlab.com/transrate/metrics.html.

assembly	EveBCdTP1_all	EveBCdTP1_ani	EcyBCdTP1_all	EcyBCdTP1_ani	GlaBCdTP1_all	GlaBCdTP1_ani
n_seqs	790,102	30,237	1,070,909	34,579	82,9471	38,650
smallest	201	201	201	201	201	201
largest	22,553	22,553	23,639	23,639	21,537	21,503
n_bases	359,353,035	34,877,874	482,300,499	39,864,864	387,496,885	43,751,838
mean_len	455	1153	450	1153	467	1132
n_over_1k	55,880	10,911	73,613	12,046	65,886	13,966
n_over_10k	92	46	93	38	76	40
n_with_orf	61,709	18,815	77,108	21,875	74,993	26,518
mean_orf_percent	57	80	56	80	58	81
n90	230	458	229	460	230	486
n70	318	1069	313	1031	323	981
n50	497	1870	488	1869	530	1673
n30	954	2889	948	2987	1051	2658
n10	2456	5158	2520	5299	2556	4894
gc	0.43	0.50	0.43	0.49	0.44	0.47

Mapping rate

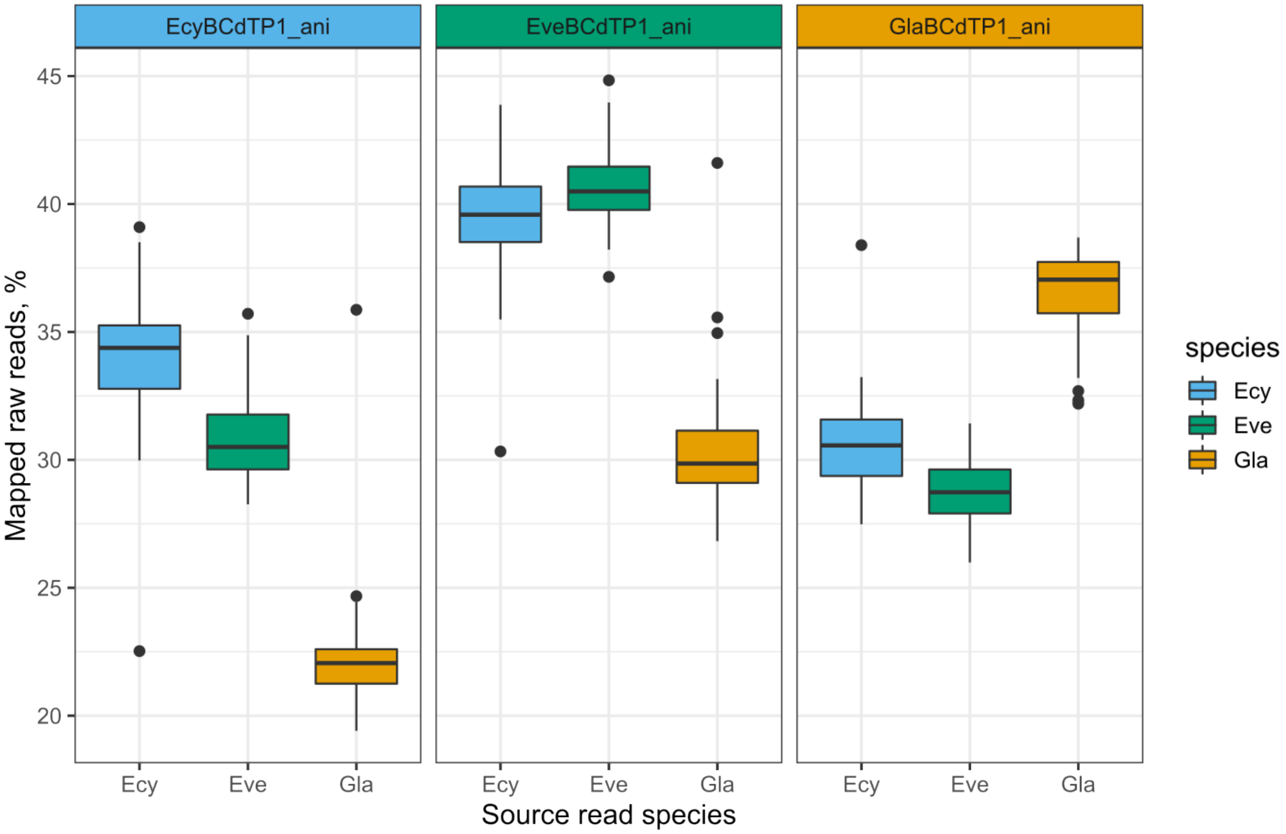

Figure S1: The percent of mapped reads was calculated by mapping raw reads of each species to each of the assemblies with salmon [47] and extracting the mapping rate from salmon output. Each box plot summarizes *ca.* 60 values.

Text S1: Checks for mislabeled samples

To check for consistency of samples and variability between replicates, we used the transcript abundance data generated by mapping of all samples to one transcriptome assembly to perform principal component analysis (PCA).

Two first principal components showed a clear distinction between the species. Moreover, while PC1 differentiates between *Eulimnogammarus* and *Gammarus*, PC2 differentiates between *E. verrucosus* and *E. cyaneus*.

However, several samples were located not in the expected places on the plot (an example is show below). A possible explanation could be that two samples have been swapped during library preparation, and one was a mixture of material in between the two species.

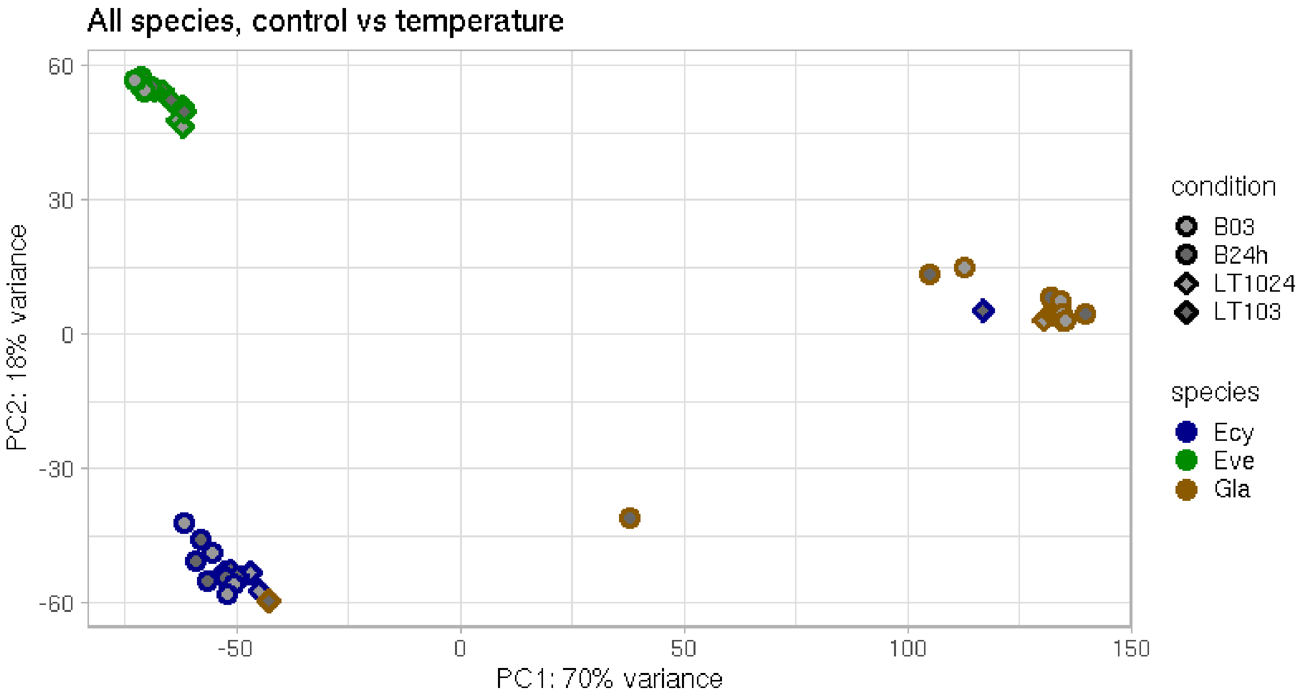

An example PCA plot of control and LT10 exposure samples showing the problematic samples. Other samples not shown on this plot were correctly attributed to the species.

To checke for potential wrong assignment of the sample to species, we analyzed 18S rRNA sequences as a phylogenetic marker. The 18S sequences of these species are known (Qiu, Y., Smith, J.E., Sherbakov, D.Y. and Kamaltynov, R.M., unpublished).

```
FJ752394.1 Eulimnogammarus verrucosus voucher EVER8 18S ribosomal RNA gene, partial sequence;
FJ752393.1 Eulimnogammarus cyaneus voucher ECYA9 18S ribosomal RNA gene, partial sequence;
FJ752398.1 Gammarus lacustris voucher GLAC1 18S ribosomal RNA gene, partial sequence
```

We aligned these sequences and then manually chose the most variable region for faster check:

```
>FJ752394.1_Eulimnogammarus_verrucosus_v
TTGGGGCTTGCTTGTCTTGC-CCTGCGCTGCTCTGACGGATGCTTTTATTAG
ACCAAGCCGCTGAGGACTTGAGGGTTCGCGCTCTCTTGTTTGACTCGTGTG
>FJ752393.1_Eulimnogammarus_cyaneus_vouc
TTTGTGCTTGCTTGTCTTGCGCTTGCGTTGCTCTGACGGATGCTTTTATTAG
ACCAAGCCGCTGAGGACTTGAGGGTTCGCGCTCTCTTGTCTGACTCGTGTG
>FJ752398.1_Gammarus_lacustris_voucher_G
GTTGTGCTTGCTTGTCTTGCGCTCTCACTGCTCTGACGGATGCTTTTATTAG
ACCAAACCGCTGAGGACTTGGGGGTT--CGCTCTCTTGTCTGACTCGTTAT
```

Then we looked up these sequnces in the raw reads with bbmap (bbduk, https://sourceforge.net/projects/bbmap/) against the three species.

Here are the data for a subset of samples (including the most "interesing" ones). The vertical scale shows number of reads with the corresponding species-specific 18S sequence in the reads:

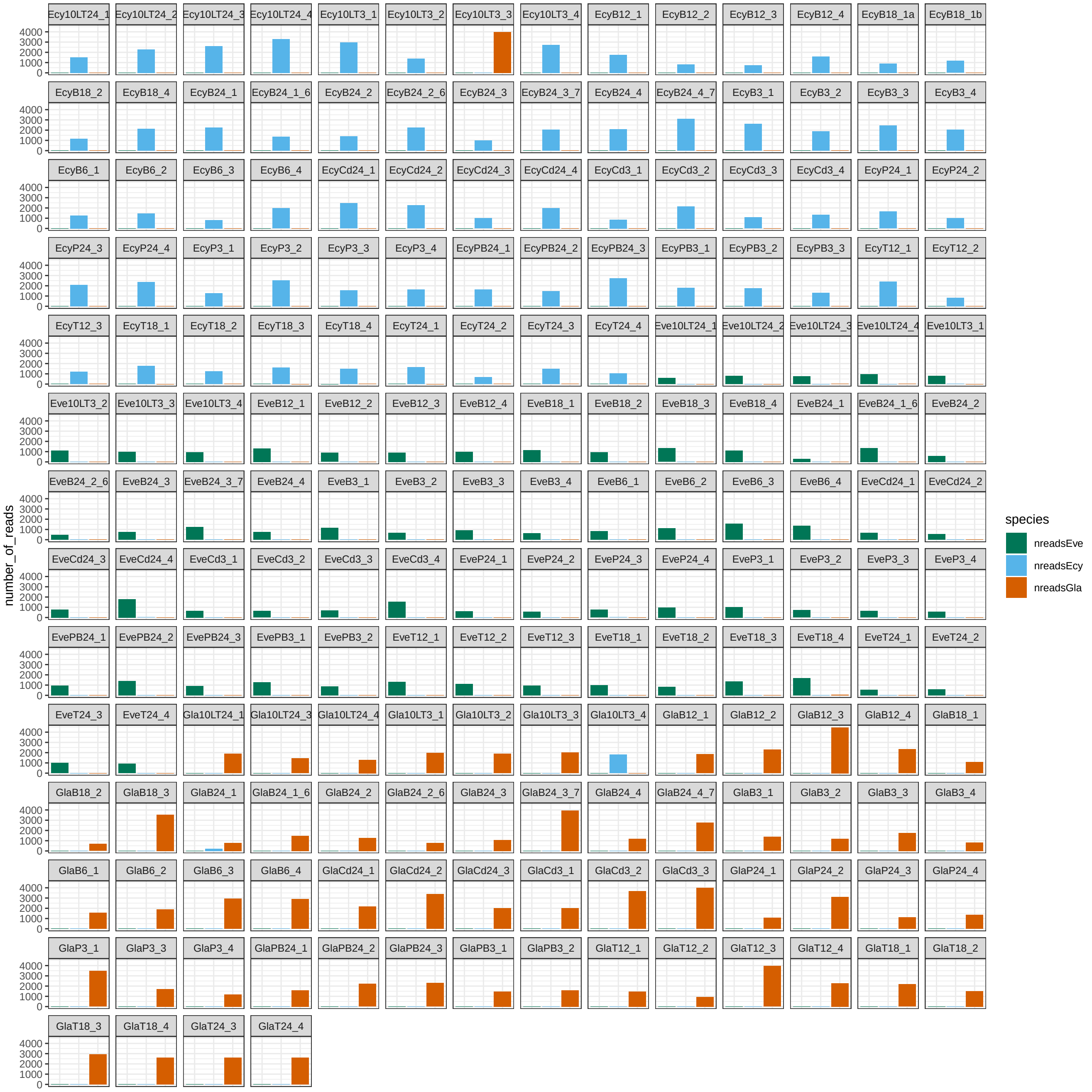

Indeed, we found two samples that have been most probably swapped and one more sample that potentially contained mixed materials of two different species. Two swapped samples were renamed, while the mixed sample was removed from further analysis. Thus, we renamed `Ecy10LT3_3` and `Gla10LT3_4` and excluded `EveB24_2_6` and `GlaB24_1` from the analysis.

Figure S2: transcripts of glutathione S transferase gene family

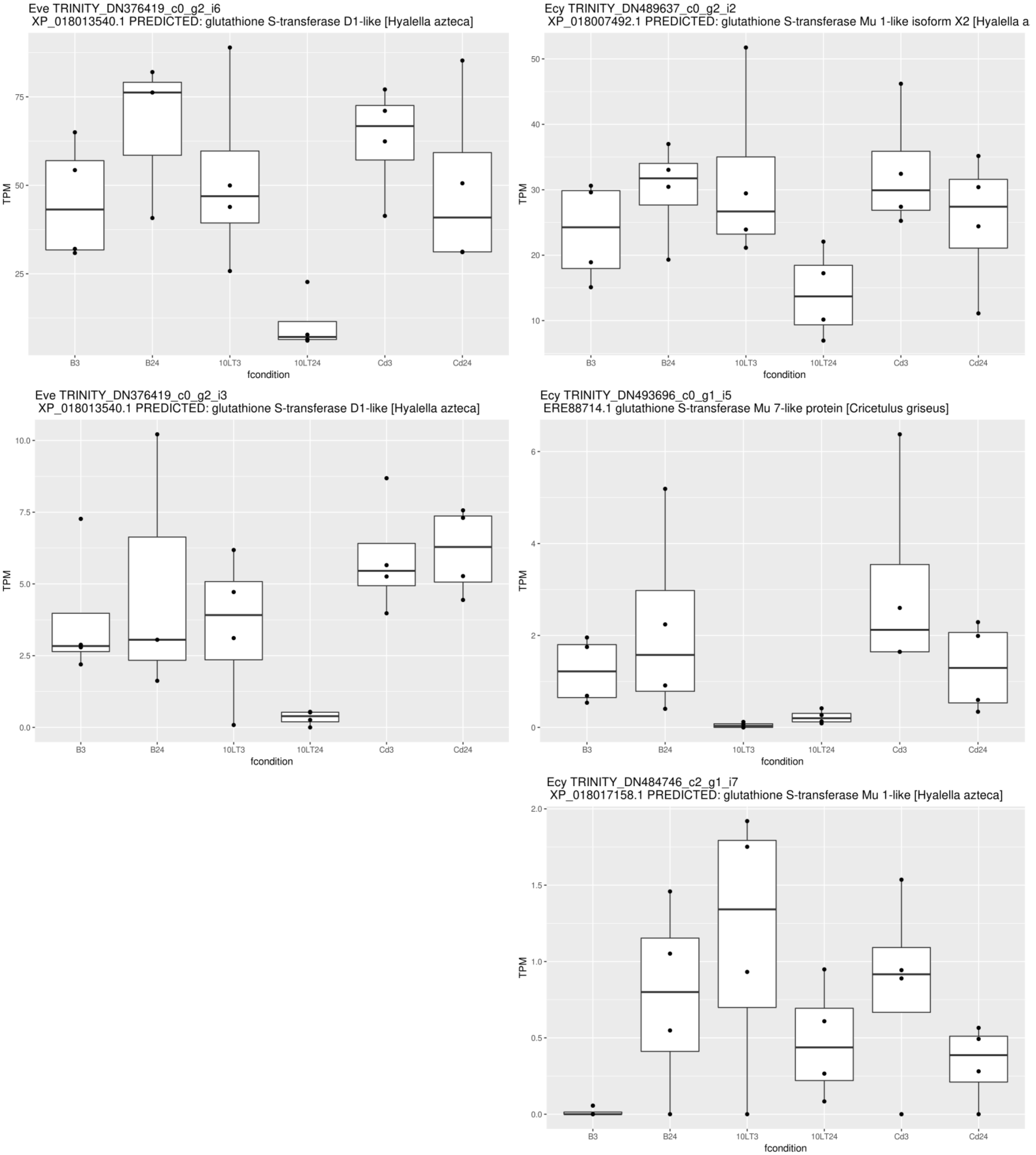

Figure S2: Abundance of different transcripts encoding glutathione S transferases in different conditions. TPM, transcript per million. The three-letter species designations, contig names and annotation are shown on the top of each plot. The conditions are listed along the horisontal axis. B3, parallel control for the 3-hr treatments. B24, parallel control for the 24-hr treatments. 10LT3, LT10 treatment for 3 hrs. 10LT24, LT10 treatment for 24 hrs. Cd3, LC10 $CdCl_2$ treatment for 3 hrs. Cd24, LC10 $CdCl_2$ treatment for 24 hrs.

Figure S3: Putative metallothionein (MT) transcripts

Figure S3: Putative MT transcripts

The upper panel features alignment of the sequences of most abundant potential MT transcripts from *E. verrucosus* and *E. cyaneus* (the same as shown in Figure 4D) with the sequence from *A. tomentosa*. Note the absolute conservation of the cystein residues. The remaining panels feature the abundance of different transcripts encoding all MT-like transcripts in different conditions. TPM, transcript for million counts. The labels of the horizontal axis are the same as in Figure S2.

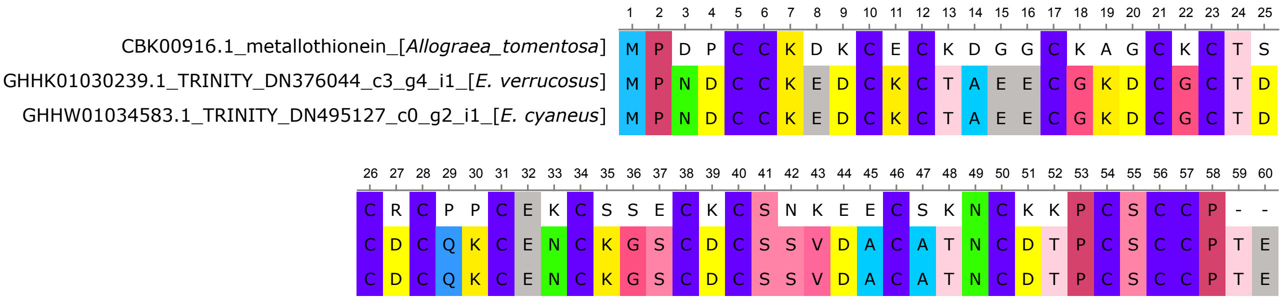

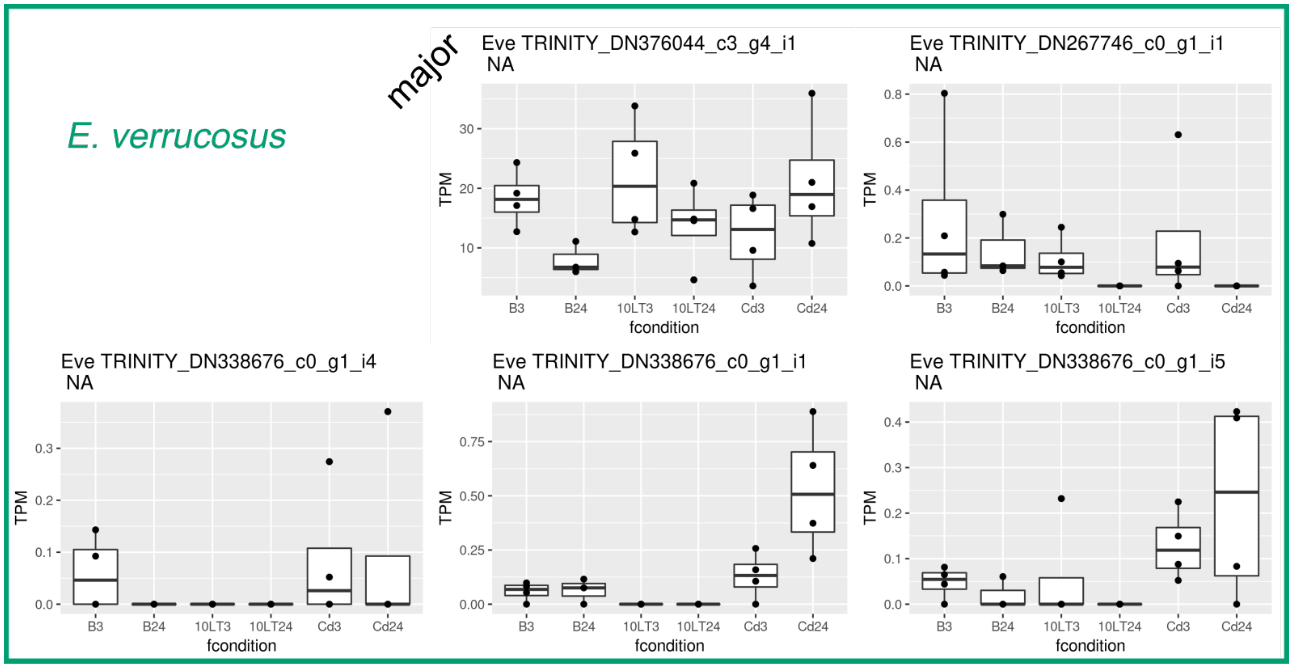

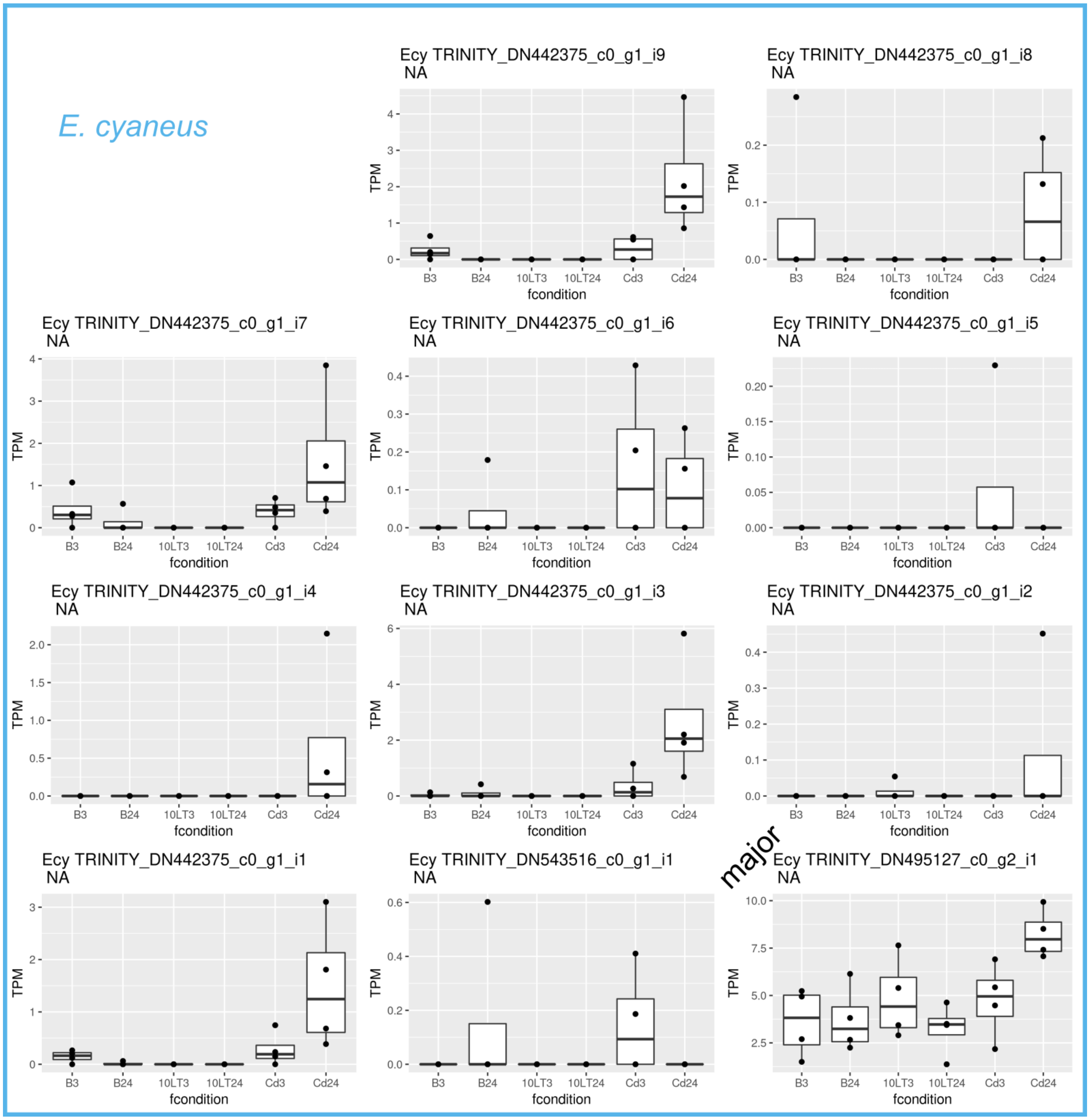
E. cyaneus
Ecy TRINITY_DN442375_c0_g1_i9
NA
Ecy TRINITY_DN442375_c0_g1_i8
NA
Ecy TRINITY_DN442375_c0_g1_i7
NA
Ecy TRINITY_DN442375_c0_g1_i6
NA
Ecy TRINITY_DN442375_c0_g1_i5
NA
Ecy TRINITY_DN442375_c0_g1_i4
NA
Ecy TRINITY_DN442375_c0_g1_i3
NA
Ecy TRINITY_DN442375_c0_g1_i2
NA
Ecy TRINITY_DN442375_c0_g1_i1
NA
Ecy TRINITY_DN543516_c0_g1_i1
NA
major
Ecy TRINITY_DN495127_c0_g2_i1
NA
TPM
fcondition
B3
B24
10LT3
10LT24
Cd3
Cd24

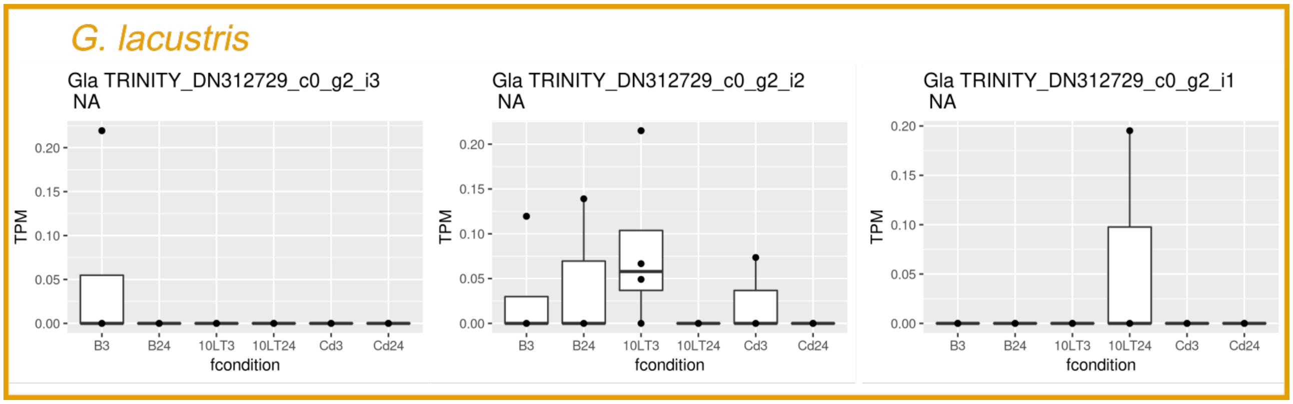
G. lacustris
Gla TRINITY_DN312729_c0_g2_i3
NA
Gla TRINITY_DN312729_c0_g2_i2
NA
Gla TRINITY_DN312729_c0_g2_i1
NA
TPM
fcondition
B3
B24
10LT3
10LT24
Cd3
Cd24
